# Development and Content Validation of the Information Assessment Method for Patients and Consumers

**DOI:** 10.2196/resprot.2908

**Published:** 2014-02-18

**Authors:** Pierre Pluye, Vera Granikov, Gillian Bartlett, Roland M Grad, David L Tang, Janique Johnson-Lafleur, Michael Shulha, Maria Cristiane Barbosa Galvão, Ivan LM Ricarte, Randolph Stephenson, Linda Shohet, Jo-Anne Hutsul, Carol A Repchinsky, Ellen Rosenberg, Bernard Burnand, France Légaré, Lynn Dunikowski, Susan Murray, Jill Boruff, Francesca Frati, Lorie Kloda, Ann Macaulay, François Lagarde, Geneviève Doray

**Affiliations:** ^1^Department of Family MedicineMcGill UniversityMontreal, QCCanada; ^2^Information Technology Primary Care Research GroupDepartment of Family MedicineMcGill UniversityMontreal, QCCanada; ^3^Department of Social MedicineFaculty of Medicine of Ribeirao PretoUniversity of Sao PauloSao PauloBrazil; ^4^School of Electrical and Computer EngineeringUniversity of Campinas - UNICAMPCampinasBrazil; ^5^Department of PsychologyUniversité du Québec à Montréal (UQAM)Montreal, QCCanada; ^6^The Centre for Literacy QuebecMontreal, QCCanada; ^7^Canadian Pharmacists AssociationOttawa, ONCanada; ^8^Institute of social and preventive medicineLausanne University HospitalLausanneSwitzerland; ^9^Department of Family Medicine and Emergency MedicineUniversité LavalQuebec, QCCanada; ^10^Library ServicesCollege of Family Physicians of CanadaLondon, ONCanada; ^11^Life Sciences LibraryMcGill UniversityMontreal, QCCanada; ^12^Health Sciences LibraryJewish General HospitalMontreal, QCCanada; ^13^Lucie and André Chagnon FoundationMontreal, QCCanada

**Keywords:** information use, information retrieval, push technology, consumer health information, consumer-centered outcomes, content validity

## Abstract

**Background:**

Online consumer health information addresses health problems, self-care, disease prevention, and health care services and is intended for the general public. Using this information, people can improve their knowledge, participation in health decision-making, and health. However, there are no comprehensive instruments to evaluate the value of health information from a consumer perspective.

**Objective:**

We collaborated with information providers to develop and validate the Information Assessment Method for all (IAM4all) that can be used to collect feedback from information consumers (including patients), and to enable a two-way knowledge translation between information providers and consumers.

**Methods:**

Content validation steps were followed to develop the IAM4all questionnaire. The first version was based on a theoretical framework from information science, a critical literature review and prior work. Then, 16 laypersons were interviewed on their experience with online health information and specifically their impression of the IAM4all questionnaire. Based on the summaries and interpretations of interviews, questionnaire items were revised, added, and excluded, thus creating the second version of the questionnaire. Subsequently, a panel of 12 information specialists and 8 health researchers participated in an online survey to rate each questionnaire item for relevance, clarity, representativeness, and specificity. The result of this expert panel contributed to the third, current, version of the questionnaire.

**Results:**

The current version of the IAM4all questionnaire is structured by four levels of outcomes of information seeking/receiving: situational relevance, cognitive impact, information use, and health benefits. Following the interviews and the expert panel survey, 9 questionnaire items were confirmed as relevant, clear, representative, and specific. To improve readability and accessibility for users with a lower level of literacy, 19 items were reworded and all inconsistencies in using a passive or active voice have been solved. One item was removed due to redundancy. The current version of the IAM4all questionnaire contains 28 items.

**Conclusions:**

We developed and content validated the IAM4all in partnership with information providers, information specialists, researchers and representatives of information consumers. This questionnaire can be integrated within electronic knowledge resources to stimulate users’ reflection (eg, their intention to use information). We claim that any organization (eg, publishers, community organizations, or patient associations), can evaluate and improve their online consumer health information from a consumers’ perspective using this method.

## Introduction

### Background

The availability of the Internet and expectations of the public to become more involved in health care decisions have resulted in unprecedented demand of consumer health information, such as that provided by the National Library of Medicine (MedlinePlus). Consumer health information is about health problems, self-care, disease prevention, and health care services, and is intended for the general public, including patients and their relatives [1,2]. Accessing Web-based health information will increase as Internet access is almost ubiquitous in many countries. For example, a national survey of a representative population sample reveals that accessing Web-based consumer health information in the United States has doubled since 2001, while access from other sources has decreased, and the Internet is the most frequently used platform for accessing consumer health information [3]. In 2012, 35% of US adults reported using health information found online “to try to figure out the medical condition they or someone else might have” [4].

Using evidence-based health information may contribute to improving the health of populations, when this information use leads consumers to improve their knowledge and their participation in health decision-making [5-8]. Another potential impact of better-informed consumers is on the use of health services. For example, in one study, better-informed consumers were less likely to use emergency room services [9]. Such outcomes are particularly important in primary health care where patients, their friends, relatives, and home care aids, play an active role in health decision-making and frequently search for information [10]. For example, among adult Internet users in Canada, 54% have looked for health information online [11]. The aging of society and the prevalence of chronic diseases will continue to increase [12], which will, in turn, drive an increase in the use of Web-based consumer health information.

In line with more consumer-centered health services, health information providers are producing more Web-based resources for the general public and for patients [13]. However, there are no comprehensive instruments to evaluate whether Web-based consumer health information is valuable from a consumers’ viewpoint. While existing tools typically focus on experts’ evaluation of the quality of information sources [14], new tools are needed to better understand the role of the Internet in health, and specifically to evaluate and improve the impact of information on patients and their families [15]. Consequently, our objective is to develop and validate a questionnaire that can be used by the general public, including patients, to assess outcomes associated with seeking and receiving Web-based consumer health information. To do this, we engaged organizational partners, such as consumer health information providers who expressed a need for a clear and user-friendly, comprehensive assessment method. This method will be used to stimulate reflection on information, enabling a two-way knowledge translation between information providers and consumers, also referred to as participatory production of knowledge with lay people. In this paper, we report on the development and validation of the Information Assessment Method for patients and consumers (IAM4all). We will present the theoretical model, the literature review, the three-step content validation process, the results, and the discussion, including examples of application and further validation study.

### Theoretical Model and Literature Review

#### Overview

The Information Assessment Method (IAM) is based on information studies [16], and a theoretical model, called the Acquisition Cognition Application - Level of Outcomes model (ACA-LO), which is presented elsewhere [17]. This model explains the value of information, (ie, how information is valuable from the user viewpoint). In this model, four levels of outcomes are associated with the retrieval or the reception of an information object (eg, a Web page): the situational relevance of the information [level 1], its cognitive/effective impact [level 2], the use of information [level 3], and subsequent health benefits [level 4]. There are four levels because situational relevance is necessary for information to have positive cognitive impact. In turn, a positive cognitive impact is necessary for applying information, which could eventually result in health benefits. The first three levels correspond to three iterative steps of human information interaction: acquisition, cognition, and application of information. Consumers may receive or retrieve Web-based health information (acquisition), understand and integrate it (cognition), and then use it (application), which may lead to health benefits. This process fits with the definition of eHealth literacy, “the ability to seek, find, understand, and appraise health information from electronic resources and apply the knowledge gained for addressing health problems” [18].

For clinicians, the ACA-LO model is operationalized using an IAM checklist that stimulates reflective learning within continuing education programs. With regard to Web-based health information, completing an IAM questionnaire will stimulate consumers’ reflection on the relevance of retrieved information for the situation at hand, the cognitive/affective impact of information (eg, learning something new), the intention to use information (eg, for doing something differently), and the expected health benefits from using it. In line with studies of the mere measurement effect [19], such reasoning is important as this process can help consumers to better reflect on information-related behaviors, which could have been otherwise overlooked. In line with the latest version of the Theory of Reasoned Action [20], a relevant information object (level 1), the positive cognitive/affective impacts of this information (level 2), the intention to use information (level 3), and expected benefits from using it (level 4) constitute key determinants of consumer behavior (information use) when information is trusted and seen as reflecting a social norm. According to this theory, two other key determinants of behavior are independent from the information object: “the person has the skills necessary to perform the behavior”, and environmental constraints do not completely block the behavior.

For the present work, we conducted a critical review of the literature (all disciplines) on the four levels of outcomes of information with regards to online consumer health information. We examined 27 articles reporting literature reviews, original studies, and doctoral theses. For each level of outcome, this review suggested a preliminary list of types of outcomes. There were (1) seven types of consumer objectives for assessing the situational relevance of information, (2) five types of cognitive/affective impact of information on consumers, (3) five types of consumer use of information, and (4) eight types of health benefits for consumers. Findings from this review were grouped in accordance with three types of information-seeking: professionally-mediated access (through clinicians and librarians), direct access, and peer-mediated access (through relatives and social media) to Web-based consumer health information. These types are complementary (eg, consumers can search by themselves and with peers, then check with a health professional). Consideration of all three types of information-seeking was important to propose a comprehensive list of types of outcomes.

#### Level 1 Outcomes: Situational Relevance (Acquisition)

##### Professionally-Mediated Access

The literature shows that consumers are provided with Web-based health information by professionals for an educational purpose. The acquisition of information is mediated by a nurse, a doctor, an allied health professional, or a librarian [21-26].

##### Direct Access

Web-based health information can be found by consumers to address the following objectives: answer their own clinical questions (about themselves or relatives); update their knowledge; find more information to complement what was provided by clinicians or librarians; check this information; and satisfy their curiosity [27-36].

##### Peer-Mediated Access

Peers usually provide Web-based health information based on personal opinions or experiences, and encouragement or emotional support (subjective); they can also provide information based on guidelines (normative), or research (objective), or a combination of subjective, normative, and objective information [31,35,37,38].

#### Level 2 Outcomes: Cognitive/Affective Impacts (Cognition)

##### Professionally-Mediated Access

A systematic literature review shows that cognitive/affective impacts of Web-based consumer health information on patients are mainly learning (patients learn something new) and reassurance for decision-making (patients feel reassured for making decisions about their health and health care) [39].

##### Direct Access

The literature suggests cognitive/affective impacts are commitment to change in health care (eg, a commitment to change in prevention or treatment of illness), learning (better understanding of health or health care), and reassurance for decision-making [28,32-34].

##### Peer-Mediated Access

An overview of the field by experts suggests cognitive/affective impacts of information from members of support groups are learning (better understanding of specific issues about health or health care), reassurance (gaining confidence), and confirmation that consumers are doing the right thing [31].

#### Level 3 Outcomes: Information Use (Application)

##### Professionally-Mediated Access

Web-based consumer health information is used by professionals to communicate with patients, and can be prescribed to patients (eg, in preparation for counseling and decision-making) [21-23,40].

##### Direct Access

Web-based consumer health information is used by people for themselves, their relatives, and friends. Direct access may lead to a consultation with a clinician to get an explanation or another opinion, and to modify health care when needed. Although rarely, using Web-based consumer health information may lead to disagreements between patients and clinicians. Clinicians may be defensive, or analyze the information with patients and guide them to high quality resources [28,32-34].

##### Peer-Mediated Access

In online support groups, the flow of information involves multiple members and moves in multiple directions; it may contain inaccuracies, but most inaccuracies are rapidly corrected by informal leaders and group members. Information from these groups may be often used to comply with health care management [31,41].

#### Level 4 Outcomes: Health Benefits

##### Professionally-Mediated Access

The use of Web-based consumer health information may be associated with an increase of patients’ participation in health care, a gain in patient satisfaction, the prevention of health disease, and health improvement such as a reduction of depression and of the level of anxiety. Interactive interventions that tailor information to users’ needs can be more effective compared with noninteractive booklet or email interventions [22-24,26,42-45].

##### Direct Access

The use of Web-based consumer health information may affect the relationships between health professionals and patients, as it may (1) augment information provided by clinicians, (2) help patients to make informed health care choices, and (3) enable shared decision-making when there are different options for health care. It may transform the traditional clinician-patient relationship (from the clinician with expertise-based power to the clinician sharing information with patients), which can be challenging for some clinicians and patients. For example, consumer health information may create frustration when cyberchondriac patients bring in lengthy printouts of Web-based information about illnesses they might have [28,30,34,35].

##### Peer-Mediated Access

There is no strong evidence regarding the positive or negative effects of Web-based consumer health information such as that from online support groups, discussion forums, and mailing lists. While online cancer support groups may help group members to cope more effectively with their disease, it has been suggested that people may rely on groups for too long, which can delay needed health care [37,38,41,46].

## Methods

### Study Design

In line with usual content validation procedures for psychometric assessments [47,48], we followed three steps that involved researchers, laypersons, and a panel of experts, respectively. These three steps are described in the “Data Collection and Analysis” section below. In step 1, we created a first version of the IAM4all questionnaire using the theoretical model, and items from the critical literature review and previous work [17]. In step 2, the second version was based on interviews with 16 laypersons. For step 3, experts contributed to produce the third version by rating all items for relevance, clarity, representativeness, and specificity. Ethical approval was obtained from the McGill University institutional review board.

### Participants

Study participants were 16 laypersons (health information consumers), and 20 experts (co-authors) from McGill University, and 3 organizational partners. First, laypersons were recruited by co-authors from their adult acquaintances. They were fluent in English or French, and had no training or experience in health sciences or information studies.

Second, our partners were the Canadian Pharmacists Association (CPhA), the College of Family Physicians of Canada (CFPC), and the Centre for Literacy Québec (CLQ). We have been conducting participatory research since 2005 with the CPhA and the CFPC for the development of an IAM checklist for clinicians (nurses, pharmacists, physicians). The CPhA and the CFPC are nonprofit professional associations. The CPhA provides recommendations for clinicians (eg, patient self-care), and is starting to provide Web-based consumer health information. The CFPC produces Web-based patient education material for family physicians and patients. The CPhA and the CFPC are interested in assessing their products. For their part, the CLQ was interested in the development and assessment of effective interventions providing Web-based consumer health information. The 3 partners helped to formulate the objectives for this work. Our partnership followed the principles of participatory research with organizations, also called collaborative action research [49,50]. This approach leads to improve knowledge and practice (organizational learning), to engage organization members (as reflective practitioners), and to involve organization partners in all research aspects (integrated knowledge translation).

### Data Collection and Analysis

#### Step I: Researchers (IAM4all Version 1)

We created the first version of the IAM4all questionnaire using the four levels of outcomes of information (ACA-LO model): (level 1) situational relevance of information, (level 2) its cognitive impact, (level 3) information use, and (level 4) subsequent health benefits. For each level, items were derived from our critical review of the literature on consumer health information. Then, we edited and revised these items using previous work on the IAM. Finally, items of the IAM4all questionnaire were then reviewed by 8 researchers who belonged to the Information Technology Primary Care Research Group (ITPCRG) at McGill University.

More than 34 papers and book chapters are published about our previous work on the IAM checklist for clinicians (Multimedia Appendix 1). This checklist systematically documents reflection on clinical information, delivered or retrieved from electronic knowledge resources. It enhances continuing education by stimulating clinicians’ reflective learning, evaluation of knowledge resources, and enables a two-way knowledge exchange between information users and information providers. Through literature reviews, qualitative, quantitative, and mixed-methods studies, we have documented the feasibility, content validity, construct validity, and substantive validity (theoretical rationale) of the IAM checklist.

#### Step II: Laypersons (IAM4all Version 2)

The first version of the IAM4all questionnaire was tested using individual interviews with 16 laypersons (VG). The level of health literacy of participants was tested using the Short Test of Functional Health Literacy in Adults (S-TOFHLA) [51]. Participants were asked to describe their experience with accessing consumer health information online. Then, they read a public health leaflet, and completed the IAM questionnaire based on the information in the leaflet. Participants were asked to read and rate the public health leaflet they felt was most relevant to them out of the following five: Nutrition Labeling, Smog and Your Health, Summer Food Safety, Sunscreens, and West Nile Virus [52] (our interview guide is available on request).

Interviews were both inductive and deductive. Participants were asked to describe the overall experience of consumers looking for health information, which enabled the emergence of new items. Then, they were asked for their feedback on the existing items. For each level of outcomes, the interviewer asked open questions (eg, “when you find health information on the Internet, how do you use it?”) followed by semistructured questions for each item (eg, “let me know if the item is clear; if it is not clear, tell me why”), and ended with an open question, “would you suggest any other items?”. All interviews were recorded and summarized for analysis. For each questionnaire item, the interviewees’ responses were interpreted (VG) as a confirmation (item is clear), or a revision (change wording, move to a different question, merge with another item), an addition (new item), or an exclusion. Additional comments related to each item were also recorded and interpreted. All interpretations were reviewed with the first author (PP), thus creating the second version of the IAM4all questionnaire.

#### Step III: Expert Panel (IAM4all Version 3)

In line with the usual definition of content validity for psychometric assessments [47], we asked an expert panel to assess the item relevance, clarity, representativeness, and specificity. In psychometric terms, our constructs and facets were the four levels of outcomes of information and IAM4all items, respectively. Using a Web-based survey, the second version of the questionnaire was reviewed by a panel of 20 experts (12 information specialists and 8 health researchers) including our partners (CFPC, CPhA, and CLQ).

All experts rated each item for relevance and clarity. To rate item relevance, experts were asked whether the item was appropriate to document the corresponding level of outcomes (eg, they were asked if the item “I used (will use) this information to do it differently” was appropriate to document the use of information). Four response options were available ranging from “I strongly agree” to “I strongly disagree”. To rate clarity (readability), four response options were offered ranging from “I strongly agree (accept this item without revision)” to “I strongly disagree (reject this item because of a major clarity issue)”.

In addition, all experts rated the representativeness and specificity of items for each level of outcomes in the ACA-LO model. To rate representativeness, participants were asked whether the items were representative of all aspects of the corresponding level of outcomes (eg, participants were asked if the six proposed items covered all aspects [or dimensions] of the level health benefits). To rate specificity, participants were asked whether the items for each level of outcomes were specific (ie, no item is redundant with another).

For all questions, response options ranged from “I strongly agree” to “I strongly disagree”. Participants were asked to justify their “disagree” and “strongly disagree” responses in a comment box. All experts answered all questions, and we calculated the proportion of expert agreement by combining the number of responses “I strongly agree” and “I agree” (n=20). We considered 66% or more as an acceptable proportion of expert agreement (at least 14 experts agreed). In addition, experts provided suggestions for addition, revision, or exclusion of items. The analysis of experts’ ratings and suggestions led us to confirm, revise, add, and remove items. In line with ecological validity, which is defined as the usability and adaptation of a tool from the users’ viewpoint [48,53], expert suggestions were not retained when they were contradicted by interviews with laypersons. This survey of a panel of experts led us to build the third version of the IAM4all questionnaire.

## Results

### Overview

For each level of outcomes, results are summarized in Tables 1-4. Each table reports all steps of the content validation procedure. All interviewees reported frequently searching for health information on the Internet (16 of 16). The majority of interviewees were women (11 of 16). In terms of health literacy, all 16 interviewees had an Adequate Functional Health Literacy level [51] (ie, can read and interpret most health texts).

**Table 1:** Content validation of IAM4all items: level 1 - situational relevance.

Post interview items	Prepanel item development					Expert panel (N=20)			
	Theoretical model	Critical review	Previous work on the IAM	Consultation with 8 researchers	Interviews with 16 laypersons	Item relevance	Clarity	Representa-tiveness	Specificity
Situational relevance	x	x	x	x	x			85%	50%
1. To answer a question about my health		x	x	x	x	100%	100%		
2. To address a question about the health of a relative or a friend		x	x	x	x	95%	90%		
3. To educate myself about health		x	x	x	x	90%	95%		
4. To satisfy my curiosity about health		x	x	x	x	80%	90%		
5. To follow-up on the information given by a health professional		x	x	x	x	100%	95%		
6. To prepare myself before talking to a health professional			x	x	x	100%	100%		
7. To make a decision about seeing a health professional					x	100%	95%		
8. To find options different from those given by a health professional					x	95%	95%		

**Table 2:** Content validation of IAM4all items: level 2 - cognitive impact.

Post interview items	Prepanel item development					Expert panel (N=20)			
	Theoretical model	Critical review	Previous work on the IAM	Consultation with 8 researchers	Interviews with 16 laypersons	Item relevance	Clarity	Representa-tiveness	Specificity
Cognitive impact	x	x	x	x	x			75%	55%
9. I learned something new		x	x	x	x	100%	100%		
10. This information confirmed I did (am doing) the right thing		x	x	x	x	100%	85%		
11. I was reassured		x	x	x	x	80%	80%		
12. I was reminded of something I already knew			x	x	x	100%	95%		
13. I am motivated to learn more					x	95%	90%		
14. I understood this information		x	x	x	x	90%	95%		
15. I was dissatisfied			x	x	x	85%	75%		
16. There is a problem with this information			x	x	x	85%	85%		
17. This information could be harmful			x		x	65%	65%		

**Table 3:** Content validation of IAM4all items: level 3 - information use.

Postinterview items	Prepanel item development					Expert panel (N=20)			
	Theoretical model	Critical review	Previous work on the IAM	Consultation with 8 researchers	Interviews with 16 laypersons	Item relevance	Clarity	Representa-tiveness	Specificity
Information use	x	x	x	x	x			55%	85%
18. I was doing or going to do something concerning my health, and I used (will use) this information to do it differently		x	x	x	x	95%	80%		
19. I did not know what to do, and this information (did) will help to justify a decision concerning my health			x	x	x	90%	80%		
20. I thought I knew what to do, and I used (will use) this information to be more certain about the management of my health (or health care)			x	x	x	85%	75%		
21. This information (did) will help to better understand a particular issue related to my health			x	x	x	90%	75%		
22. I used (will use) this information in a discussion with a health professional		x	x	x	x	100%	95%		

**Table 4:** Content validation of IAM4all items: Level 4 - expected benefits.

Postinterview items	Prepanel item development					Expert panel (N=20)			
	Theoretical model	Critical review	Previous work on the IAM	Consultation with 8 researchers	Interviews with 16 laypersons	Item relevance	Clarity	Representa-tiveness	Specificity
Expected benefits	x	x	x	x	x			60%	60%
23. This information decreased my worries about a health problem		x	x	x	x	90%	85%		
24. This information increased my satisfaction with the care I receive		x	x	x	x	90%	90%		
25. This information allowed (will allow) me to receive additional information from a health professional		x	x	x	x	75%	70%		
26. Because of this information, I am (will be) more involved in decisions around my health		x	x	x	x	100%	90%		
27. This information helped (will help) me to better handle a problem with my health		x	x	x	x	100%	90%		
28. This information helped (will help) me to prevent a disease or the worsening of a disease		x	x	x	x	85%	80%		
29. This information helped (will help) to improve my health		x	x	x	x	100%	85%		

### Step I: IAM4all Version 1

The ACA-LO model provided the four interdependent levels of outcomes of information targeted by the IAM4all questionnaire. Findings of our critical review suggested 25 items to operationalize these levels. There were (1) seven types of consumer objectives for the situational relevance of information, (2) five types of cognitive/affective impact of information on consumers, (3) five types of consumer use of information, and (4) eight types of health benefits for consumers. The consultation with 8 researchers yielded the first version of the questionnaire that included 26 items.

### Step II: IAM4all Version 2

The analysis of interviews with 16 laypersons resulted in 18 items being confirmed, six revised, three excluded, and five added. All six revisions involved rewording of the questionnaire items. Interviewees suggested excluding the reason for searching item “to share information about health with members of a support group” and the information use item “I used (will use) this information to persuade a health professional to make a change in the management of my health” as they were not representative of the consumer experience. Interviewees brought to our attention two new reasons for searching: “To make a decision about seeing a health professional”, and “to find options different from those given by a health professional”. The three other added items reflected the interviewees experience with finding information that motivated them to learn more, using the found information to be more certain about the management of their health (or health care), and information being harmful. Six of 16 interviewees (37.5%) mentioned that retrieved information often increased their anxiety about health issues, and 2 (12.5%) said that information could be harmful:

I tend to over-worry, I tend not to look up for too much information and prefer to talk to the doctor about it. The more information I have the more I will worry.

P02

Another interviewee shared this feeling by saying:

Seldom, information on the Internet decreases your worries, it adds to the anxiety.

P03

To address this aspect using information, the item “this information made me more worried” was excluded as a cognition item and transformed as a new health benefit question: “Did something negative come out from using this information?” This analysis resulted in the second version of the questionnaire, which included 29 response items.

### Step III: IAM4all Version 3

The analysis of experts’ ratings and suggestions resulted in nine items being confirmed, 19 revised (reworded), zero added, and one removed. Regarding the relevance and clarity of each item, expert agreement was satisfactory for 28 of 29 items (96.6%). Agreement was not satisfactory for the item “the information could be harmful”. Seven experts found this item problematic (eg, 2 suggested that the user would not be able to determine if the information is harmful). However, interviewees did not perceive this item to be problematic. In fact, 2 reported that this item is important. Thus, in line with ecological validity, the users’ viewpoint was prioritized, and the item was kept. Using experts’ comments, the wording was nevertheless improved to read “I think this information could be harmful”.

Representativeness and specificity of items per level of outcomes were discussed. For each level, expert comments were addressed as follows. Level 1: the item “to educate myself” was seen as redundant with the item “to satisfy my curiosity”, because 9 experts considered curiosity as the motivation that encompasses the will to educate oneself. As a result, the item “to educate myself” was excluded. Level 2: The item “I understood this information” was transformed into a response to a new screening question, “did you find the information you were looking for?”: “Yes but I did not understand it”. Level 3: The only changes consisted of rewording three items. Level 4: Five experts were concerned about the lack of negative types of outcomes. To address this issue, the question “did something negative come out from using this information?” was added with Yes/No response options and a comment box for those who answered yes.

Finally, the wording of 19 items and their readability for users with a low level of literacy have been improved, and inconsistencies in using a passive or active voice have been solved. For example, the item “to address a question about the health of a relative or a friend” was changed to “to answer a question about the health of someone else (eg, a family member)”. As another example, the concept of “health professional” has been explained using a parenthesis with examples (a nurse, a doctor, a pharmacist, or other clinician).

## Discussion

### Principal Findings

**Figure 1:**
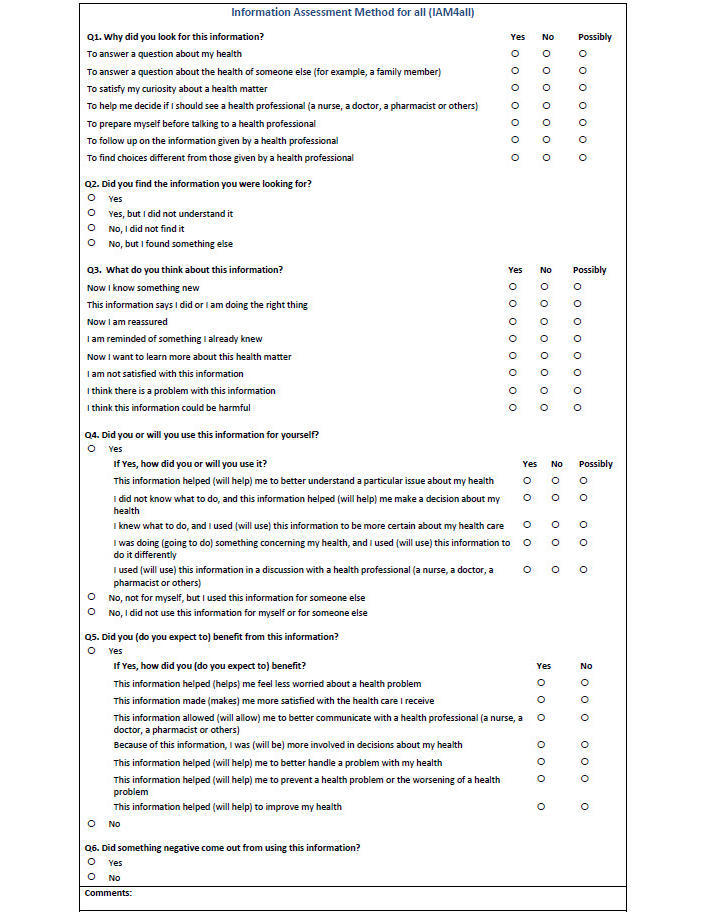
Information Assessment Method for all (IAM4all).

We developed and content-validated a questionnaire that can be used to assess four levels of outcomes associated with seeking and receiving Web-based consumer health information. For information seeking situations (pull: active acquisition of information), the current version of the IAM4all questionnaire is presented in Figure 1. The verb tense of questions and items must be adapted to the moment of the evaluation. For example, the future tense in the fourth question (will you use this information?) is appropriate for quasimomentary assessment (intention to use), while the past tense (did you use this information?) is appropriate for follow-up evaluation (self-reported use). In addition, we adapted this questionnaire for situations where information is received (push: passive acquisition of information) such as email alerts (push questionnaire available on request). To our knowledge, this validation study is the first to propose such practical method to evaluate how Web-based consumer health information objects are valuable from a consumer perspective, whatever the type of access to information (professionally-mediated, direct, peer-mediated), for pull and push situations, and for quasimomentary and follow-up evaluations.

The proposed questionnaire flow is as follows: (1) the title of the information object under scrutiny is displayed (eg, Web page title), (2) the last four questions are disabled when the answer to question #2 is “yes but I did not understand it” or “no” (did you find the information you were looking for?), and (3) the last two questions are disabled when the answer to question #4 is “no” (did you or will you use this information for yourself?). Negative answers to question #2 refer to information that is not relevant in the situation at hand. In accordance to our model, there is no level 1 outcome, which precludes levels 2, 3, and 4 outcomes (cognitive impact, information use, and health benefits). Negative answers to question #4 refer to information that is relevant, but not used. In accordance to our model, there is no level 3 outcome, which precludes level 4 outcome (health benefits).

Based on our literature review and the interviews with laypersons the following subitems can be displayed when there are negative answers to question #2 (information not relevant): the information was difficult to find; the search engine was difficult to use; there was too much information to look over; I did not have enough time; my Internet connection was slow; there was no information in a language I am comfortable in; it was hard to tell what information to trust. In addition, question #6 allows consumers to share their concerns with information use (can something negative come out from using this information?) since the question #5 may be seen as too positive (expected health benefits).

We claim that any organization wishing to evaluate and improve their Web-based consumer health information from a consumer’s perspective can use this method. By way of illustration, we are planning a project in partnership with Défibami (an association for patients living with cardioverter-defibrillators). The association emails a monthly newsletter to their members, and is interested to know how this information is valuable for their users. Members will be asked to read the main article of the newsletter, and rate it using the push IAM4all questionnaire (version named IAM4patient, available on request) as a contribution to the improvement of the information provided by their association.

Another example is a two-way knowledge translation project that we are planning with the Chagnon foundation that produces “Naître et grandir” (N&G). The objective of N&G are to inform parents and increase social awareness of the importance of early childhood development in order to create conditions and environments that are conducive to educational success. N&G comes in a variety of formats, including a website, a magazine, and a communication initiative. N&G also circulates content and maintains contact with parents through a free, personalized weekly newsletter and social networks like Facebook and Twitter. Specifically, about 100,000 families receive the weekly newsletter that gives access to a highlighted Web page from where the public can browse other pages, thus creating a push-pull system and opportunities for serendipitous information retrieval. For part of this collaboration, a button will be available on each page offering access to the IAM4all questionnaire (version named IAM4parents, available on request). The reader’s trajectory will be tracked. Users accessing Web pages from the newsletter will be offered a push-pull version of the IAM4all questionnaire, for which the first question was revised (“why did you look for this information?” was replaced by “why did you read this information?”). Users accessing Web pages from searching the Internet will be offered the pull version of the IAM4all questionnaire.

This project has already contributed to the development of the IAM4all questionnaire in three ways: the production of a push-pull version, a cross-cultural adaptation in French, and an adaptation for a broader topic (the well-being of children and their parents). The word “health” was replaced by “child” for all questions and items, and the sentence “improve my health” was replaced by “improve the well-being or health of my child” (fifth question). Based on the N&G experience with a Web-based survey, no difficulties are expected with regard to the number of responses due to the sense of community among information users (13,000 responses were obtained from an N&G survey in the past). In sum, the IAM4all questionnaire is expected to: stimulate N&G users’ reflection, thus increase information use (mere measurement effect) [19], and continuously collect constructive feedback from the N&G users, which can be used by N&G providers to improve their information services.

The IAM4all is theory-driven, and unique in terms of comprehensiveness and content validity of items. However, it constitutes only a proposal, which needs to be further validated using statistical analysis of data collected from a larger sample (construct validity). Our content validation study faces one main limitation. The participants constituted a convenience sample of Web-based health information consumers, and were not representative of the general public in terms of demographic, educational, and sociocultural characteristics (eg, they had an adequate functional health literacy level). The implementation of the IAM4all with Défibami and N&G will allow us to conduct focus groups with diverse health information consumers for further content ecological validation study [54], and collect hundreds of completed IAM4all questionnaires for further construct validation study using classical test theory and/or item response theory.

By active involvement throughout the development of this innovative information assessment method, our partners ensured that it took their needs into account, which may improve its use. This method can respond to the needs of information providers because it can document the consumer information interaction, and enable a two-way knowledge translation between information providers and information end-users. The former updates and delivers the best available information. The latter assesses this information, and can submit constructive feedback. In turn, providers may use this feedback to improve their services, which is beneficial for all parties. Such two-way knowledge translation process constitutes a participatory production of knowledge with lay people (eg, N&G with IAM4all).

### Conclusions

To our knowledge, the IAM4all is a unique and original method to assess how Web-based consumer health information is valuable from the consumers’ viewpoint, specifically the use of information and its expected health benefits. The integration of the IAM4all within electronic knowledge resources can help information providers to evaluate and improve their Web-based consumer health information services. The IAM4all can stimulate reflection and feedback about health information; this feedback can then be used to improve information services.

## Multimedia Appendix 1

The Information Assessment Method - published papers.

## Abbreviations

ACA-LO model: Acquisition Cognition Application - Level of Outcomes model
CFPC: College of Family Physicians of Canada
CLQ: Centre for Literacy Québec
CPhA: Canadian Pharmacists Association
IAM: Information Assessment Method
IAM4all: Information Assessment Method for all
ITPCRG: Information Technology Primary Care Research Group
N&G: Naître et grandir
S-TOFHLA: Short Test of Functional Health Literacy in Adults
