# Supplementary material for: Development and Content Validation of the Information Assessment Method for Patients and Consumers
Source: JMIR Res Protoc. 2014 Feb 18;3(1):e7. doi: 10.2196/resprot.2908 (PMC3958673; doi:10.2196/resprot.2908)
Supplement: Supplementary file 1 [file resprot_v3i1e7_app1.pdf]

Information Assessment Method
www.mcgill.ca/iam

Published papers

Grad RM, Pluye P, Repchinsky C, Jovaisas B, Ricarte I, Galvao C, Shulha M, Bonar J, Marlow B, Moscovici J. Physician assessments of the value of therapeutic information delivered via email. Canadian Family Physician 2014 (in press).

Pluye P, Grad RM, Repchinsky C, Jovaisas B, Lewis D, Tang DL, Granikov V, Bonar J, Marlow B. 'Better than best' evidence? The Information Assessment Method can help information providers use family physicians' feedback for 2-way knowledge translation. Canadian Family Physician 2014 (in press).

Pierre Pluye, Roland M Grad, Janique Johnson-Lafleur, Vera Granikov, Michael Shulha, Bernard Marlow, & Ivan Ricarte. The Number Needed to Benefit from Information (NNBI): Proposal from a mixed methods research study with practicing family physicians. Annals of Family Medicine 2013; 11:498-499

Bindiganavile Sridhar S, Pluye P, Grad RM. In Pursuit of a Valid Information Assessment Method for Continuing Education: A Mixed Methods Study. BMC Medical Education 2013; 13:137

Ebell M, Grad RM. Top 20 Research Studies of 2012 for Primary Care Physicians.Am Fam Physician 2013 Sep 15;88(6):380-386.

Galvao C, Ricarte I, Grad RM, Pluye P. The Clinical Relevance of Information Index (CRII): Assessing the relevance of health information to clinical practice. Health Information and Libraries Journal 2013;30:110-20.

Pluye P, Grad RM, Repchinsky C, Jovaisas B, Johnson-Lafleur J, Carrier ME, Granikov V, Farrell B, Rodriguez C, Bartlett G, Loiselle C & Légaré F. Four levels of outcomes of information-seeking: A mixed methods study in primary health care. Journal of the American Society for Information Science and Technology 2013;64(1):108-125.

Goodman K, Grad RM, Nowacki A, Hickner J. Impact of Knowledge Resources Linked to an Electronic Health Record on Frequency of Unnecessary Tests and Treatments. Journal of Continuing Education in the Health Professions 2012;32(2):108-115.

Pluye P, Grad RM, Granikov V, Theriault G, Frémont P, Burnand B, Mercer J, Marlow B, Arroll B, Luconi F, Légaré F, Labrecque M, Ladouceur R, Bouthillier F, Bindiganavile Sridhar S & Moscovici J. Feasibility of a knowledge translation CME program: Courriels Cochrane. Journal of Continuing Education in the Health Professions 2012;32(2);134-141.

Budzinski JW, Farrell B, Pluye P, Grad RM et al. An Online Knowledge Resource and Questionnaires as a Continuing Pharmacy Education Tool to Document Reflective Learning. American Journal of Pharmaceutical Education 2012;76(5):Article 82.

Grad RM, Pluye P, Vandal AC, Moscovici J, Marlow B, Shortt SED, Hill L, Bartlett G, & Goldstine I. Continuing Medical Education outcomes associated with e-mailed synopses of research-based clinical information: A research proposal. McGill Family Medicine Studies Online 2012;07:e06.

Ebell M, Grad RM. Top 20 Research Studies of 2011 for Primary Care Physicians. American Family Physician 2012;86(9):835-40.

Grad RM, Pluye P et al. Do Family Physicians Retrieve Synopses of Clinical Research Previously Read as Email Alerts? Journal of Medical Internet Research 2011;13(4): e101.

Pluye P, Grad RM, Shulha M et al. Using electronic knowledge resources for person centered medicine – I: An evaluation model. International Journal of Person Centered Medicine 2011;1(2);385-394

Pluye P, et al. Using electronic knowledge resources for person centered medicine - II: The Number Needed to Benefit from Information. The International Journal of Person Centred Medicine 2011;1(2): 395-404.

Grad RM, Pluye P, Granikov V et al. Physicians' assessment of the value of clinical information: Operationalization of a theoretical model. Journal of the American Society for Information Science and Technology 2011;62(10):1884-1891.

Bindiganavile Sridhar S. (2010). In pursuit of a valid Information Assessment Method. McGill Family Medicine Studies Online 06:e01. http://mcgill-fammedstudies-recherchemedfam.pbwiki.com/MFMSO200904e01#. Archived by WebCite® at http://www.webcitation.org/5z5i6MGoH

Pluye P, Grad RM, Johnson-Lafleur J, Bambrick T, Burnand B, Mercer J, et al. Evaluation of email alerts in practice: Part 2 – validation of the Information Assessment Method (IAM). J Eval Clin Pract 2010;16(6):1236-43.

Pluye P, Grad RM, Granikov V, Jagosh J, Leung KH. Evaluation of email alerts in practice: Part 1 - review of the literature on Clinical Emailing Channels. J Eval Clin Pract 2010;16(6):1227-35. DOI : 10.1111/j.1365-2753.2009.001301.x

Leung KH, Pluye P, Grad RM & Weston C. A reflective learning framework to evaluate CME effects on practice reflection. Journal of Continuing Education & the Health Professions 2010;30(2):78-88.

Grad RM, Pluye P,Granikov V, Johnson-Lafleur J. Many family physicians will not manually update PDA software: an observational study. Informatics in Primary Care 2009;17(4):225-30.

Pluye P, Grad RM, Repchinsky C, Farrell B, Johnson-Lafleur J, Bambrick T et al. (2009). IAM: A comprehensive and systematic information assessment method for electronic knowledge resources. In A. Dwivedi (ed.), Handbook of Research on IT Management and Clinical Data Administration in Healthcare (chapter XXXIII). Hershey: IGI Publishing

Wang R, Bartlett G, Grad RM, Pluye P. The Cognitive Impact of Research Synopses on Physicians. Informatics in Primary Care 2009;17(2);79-86.

Mysore N, Pluye P, Grad RM & Johnson-Lafleur J. Tensions associated with the use of electronic knowledge resources within clinical decision-making processes. International Journal of Medical Informatics 2009;78:321-329.
Grad RM, Pluye P, Mercer J, Marlow B, Beauchamp ME, Shulha M, Johnson-Lafleur J, Dauphinee SW. Impact of Research based Synopses Delivered as Daily email: A Prospective Observational Study. J Am Med Inform Assoc 2008;15(2):240-45.

Pluye P, Grad RM, Mysore N, Knaapen L, Johnson-Lafleur J & Dawes M. Systematically Assessing the Situational Relevance of Electronic Knowledge Resources: A Mixed Methods study. JAMIA 2007;14:616-25.

Pluye P, Grad RM, Dawes M & Bartlett JC. Seven reasons why family physicians search clinical information-retrieval technology: Toward an organizational model. Journal of Evaluation in Clinical Practice 2007;13(1):39-49.

Grad RM, Pluye P, Hanley J, Marlow, B, Macaulay A & Dalkir K. Validation of a Method to Assess the Impact of Electronic Knowledge Resources on Clinicians. e-Service Journal 2007;5(2):e113. http://dx.doi.org/10.2979/ESJ.2007.5.2.113.

Pluye P & Grad R. Cognitive impact assessment of electronic knowledge resources: A mixed methods evaluation study of a handheld prototype. AMIA 2006 Symposium Proceedings: 634-638.

Pluye P, Grad RM, Dunikowski L & Stephenson R. The Impact of Clinical Information-Retrieval Technology on Physicians: A Literature Review of Quantitative, Qualitative and Mixed-method Studies. International Journal of Medical Informatics 2005;74(9):745-768.

Pluye P, Grad RM, Stephenson R & Dunikowski L. A new impact assessment method to evaluate knowledge resources. AMIA 2005 Symposium Proceedings: 609-613.

Grad RM, Pluye P, Meng Y, Segal B & Tamblyn R. Assessing the impact of clinical information-retrieval technology in a Family Practice Residency. Journal of Evaluation in Clinical Practice 2005;11(6):576-586.

Pluye P & Grad RM. How information retrieval technology may impact on physician practice: An organisational case study in family medicine. Journal of Evaluation in Clinical Practice 2004;10(3), 413-430.
